# Epidemiological Association Between Long-Term Fine Particulate Matter Exposure and Cardiovascular Disease: A Systematic Review and Meta-Analysis

**DOI:** 10.3126/nje.v15i4.88662

**Published:** 2025-12-31

**Authors:** Nimra Nabi, Rutbah Amin Khairati, Zaira Fatima, Umar Arif, Waleed Iqbal, Umair Abrar, Abdullah Tariq, Jahanzaib Awan, Muhammad Hammad Ul Haq, Mirza Muhammad Hadeed Khawar, Muneeb Khawar

**Affiliations:** 1 Karachi Institute of Medical Sciences, Karachi, Sindh, Pakistan; 2 George Eliot Hospital, Nuneaton, United Kingdom; 3 Bahria University Health Sciences, Karachi, Sindh, Pakistan; 4 Ninewells Hospital, Dundee, United Kingdom; 5 Orthopaedic and Medical Institute, Karachi, Sindh, Pakistan; 6 Al-Khidmat Hospital, Sahiwal, Punjab, Pakistan; 7 Islamic International Medical College, Rawalpindi, Punjab, Pakistan; 8 Liaquat College of Medicine and Dentistry, Karachi, Pakistan; 9 Services Institute of Medical Sciences, Lahore, Pakistan; 10 King Edward Medical University, Lahore, Pakistan

**Keywords:** Particulate Matter, Cardiovascular Disorders, Mortality, Air Pollution

## Abstract

**Background:**

Air pollution and particularly the fine particulate matter (PM2.5) is a major environmental risk factor of cardiovascular diseases (CVD). Millions of untimely deaths every year have been reported because of it. The epidemiological literature has linked long-term exposure to PM2.5 and mortality rates of CVDs. This meta-analysis aims to synthesize the estimates of long-term PM2.5 exposure and CVDs.

**Methods:**

Based on the PRISMA guidelines, PubMed, Embase, Web of Science, and Scopus were searched up to October 2025. Random-effects model was used to pool HRs, and the heterogeneity was measured with the help of I^2^.

**Results:**

Four studies were included in this meta-analysis. There was an increased risk of CVD with higher exposure to PM2.5 on a long-term basis (pooled HR=1.22, 95% CI: 1.06–1.41; p=0.006; I^2^=96%). Cardiovascular mortality did not show any significant association with PM2.5 exposure (pooled HR=1.00, 95% CI: 0.71–1.41; p=0.98; I^2^=29%). No significant difference was found between the PM2.5 exposure and ischemic heart disease (IHD) (pooled HR=1.65, 95% CI: 0.90–3.00; p=0.10; I^2^=95%). The same pattern was noted between the PM2.5 exposure and stroke (pooled HR=1.61, 95% CI: 0.96–2.68; p=0.07; I^2^=74%).

**Conclusion:**

The PM2.5 exposure is associated with high CVD in the long term. Results reveal the significance of establishing strict air-drome quality standards and targeted interventions to mitigate the risks in the areas of issues. More integrated studies are required to support our findings and fill the knowledge gap

## Introduction

One of the most dangerous hazards to human health in any region of the globe is air pollution. It has caused an estimated 7 million premature deaths annually with cardiovascular diseases (CVD) [1]. Particulate Matter (PM) with Air Exchange Rate of 2.5micrometers (or less) is particularly lethal since it can penetrate the bloodstream and can be absorbed in the bloodstream to cause damage. This type of infiltration leads to systemic inflammation, oxidative stress, and endothelial dysfunction that are among the key pathways to atherosclerosis, hypertension, myocardial infarction, and stroke [2]. The epidemiological evidence has demonstrated that short and long-term exposure to PM2.5 is associated with CVDs morbidity and mortality, and the high risk even exists at levels that are below the current levels of regulation [3]. Since it has been known that high-pollution areas are related to exposure to PM2.5 and the greater level of PM2.5 is associated with cardiovascular events, it has been stated that PM2.5 is a modifiable risk factor of the CVD [4].

The mechanism through which PM 2.5 leads to CVD is vascular damage. The chronic exposure of PM 2.5 is one of the main contributors to the instability of atherosclerotic plaque and it promotes the occurrence of acute events. This threat is particularly affecting vulnerable groups including the elderly [5]. As the world is becoming more urbanized, it is important to explain the long-term effects of PM2.5 on CVD. The latest epidemiological research on long-term PM2.5 and CVD is based on the large cohort studies which combine geospatial modeling analysis and satellite data with ground-based monitors to estimate exposure one-by-one [6]. Examples of studies in which Cox proportional hazards models were applied to estimate the hazard ratio are Kerman Permanente cohort and multi-national registries. A meta-analysis has also shown that a 10 ug/m^3^ PM 2.5 exposure increases the risk of IHD, stroke, and the dose-response curve is often linear [7]. The advanced technological processes, e.g., the ensemble models and the time sensitive evaluations have helped in the accurate estimation of these hazards in terms of time and space [8]. Still, there are a lot challenges. These advanced methods do not account for the people who keep migrating from one place to another and that’s why they don’t give accurate estimates. Social and economic status (SES) and existing health problems can also confuse the results, especially in regions with limited data such as Asia and the Middle East. Thus a comprehensive analysis is necessary that can guide the policy makers to make amendments in the policies and pay close attention to this serious matter.

This meta-analysis aims to do a systematic synthesis and quantification of the epidemiological evidence on the association between the long-term exposure to PM 2.5 and the CVD outcomes. The focus will involve global data, and it will help in making evidence-based interventions in air quality.

## Methodology

This meta-analysis is written as per the Preferred Reporting Items of meta-analyses and systematic reviews (PRISMA) guidelines [9].

### Eligibility Criteria

The inclusion criteria were the adults (age 15 years and older) without previous cardiovascular events at baseline and having long-term exposure to PM2.5, in terms of annual mean concentration or other measures of chronic exposure. Animal studies, reviews, abstracts, editorials and studies that failed to provide adjusted HRs or confidence intervals (CIs) were excluded.

### Information Sources and Search Strategy

To find the relevant articles, PubMed/MEDLINE, Embase, Web of Science, and Scopus were searched since the inception until October 20, 2025. MeSH terms and free-text words related to PM 2.5 exposure and cardiovascular outcomes were combined into the search strategy. The PubMed search strategy was as follows: (PM2.5" OR PM 2.5" OR fine particulate matter OR ambient air pollution) AND ((long-term exposure) OR chronic exposure OR prolonged exposure) AND (cardiovascular disease) OR CVD OR heart disease OR ischemic heart disease OR myocardial infarction OR stroke OR cerebrovascular disease OR cardiovascular mortality).

### Study Selection

Two independent reviewers were employed to screen titles and abstracts using Zotero software. Disagreement was settled through a talk with a third reviewer. Reasons for exclusion were also noted.

### Data Collection

The data were extracted on a piloted extraction sheet by two reviewers working independently. Differences of opinion were settled upon a discussion with the third reviewer. We could extract the following in each of the studies: Study (Year), Country / Region, Study Design, Study Population, Sample Size, Age Range / Mean Age, Follow-up Duration, PM2.5 Exposure Assessment, Cardiovascular Outcomes.

### Risk of Bias Assessment

The Newcastle-Ottawa Scale (NOS) was used to conduct the risk of bias assessment [10]. The following are the ratings given on this scale based on the following criteria: selection (maximum of 4 stars), comparability (maximum of 2 stars) and outcome (maximum of 3 stars). The studies were graded by two reviewers, and the areas of disagreement were resolved by discussions. Low risk of bias were indicated by a score of 7 or above.

### Statistical Analysis

Statistical analysis was done in Review Manager (RevMan) version 5.4. Random effects model was used for all outcomes. We used the inverse-variance method to pool HRs where at least two studies provided similar data with respect to each outcome (CVD, Cardiovascular mortality, IHD, stroke). To determine heterogeneity, I^2^ statistic (>50% = high heterogeneity) and Cochrane Q test (P <0.10 = significant heterogeneity) were used. Heterogeneity sources were identified through the leave-one-out sensitivity analyses. No formal test of publication bias (i.e. using funnel plots or the Egger test) was performed because there were less than 10 studies per outcome. P < 0.05 was considered statistically significant.

## Results

### Study Selection

**Figure 1:**
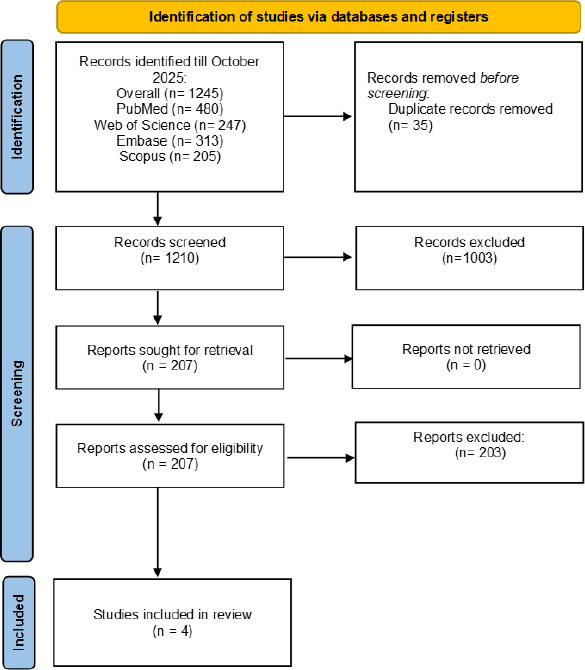
PRISMA flowchart

The systematic literature search found 1,245 records from the databases. 35 duplicate records were found and therefore 1,210 records were screened using titles and abstracts. Among them 1,003 were eliminated based on failure to satisfy the inclusion criteria, mainly because of a non-cohort study design, short-term exposure assessment, or most common irrelevant outcomes. The rest 207 articles were reviewed in full-text eligibility. After careful review, 203 studies were excluded that lead to inclusion of four [3, 11-13] cohort studies in the end. Figure 1 (PRISMA flow diagram) provides a summary of the study selection process.

### Characteristics of Included Studies

Four cohort studies that were used in this meta-analysis were carried out in China (Northwestern regions and Rural regions), the United States (California), and Denmark and involved an approximate number of 9.6 million people. Two studies were based in China, one in a large community-based set of adults with no baseline level of CVD in the northwestern regions and another one in rural residents of multicenter cohort and the rest involved a large U.S. general population of adults (Kaiser Permanente members) and Danish older adults in national screening trials. The sample size (15,502 to 5.8 million) and the follow-up of the participants (3 to 10 years) were extracted. All the studies were based on cohort design (one retrospective and three prospective) and utilized validated and high-resolution models to estimate long-term PM2.5 exposure. Medical records or national registries were used to determine cardiovascular outcomes. Each of the studies corrected major demographic, lifestyle, and socioeconomic confounders. A brief overview of the study characteristics is given in Table 1.

### Quality assessment

The quality of the included studies was determined based on NOS scale. The quality of all four articles was considered to be high, with the overall rating between 7 and 9 stars, which implies the low risk of bias in all the domains assessed (Table 2).

### Clinical Outcomes

**Figure 2.**
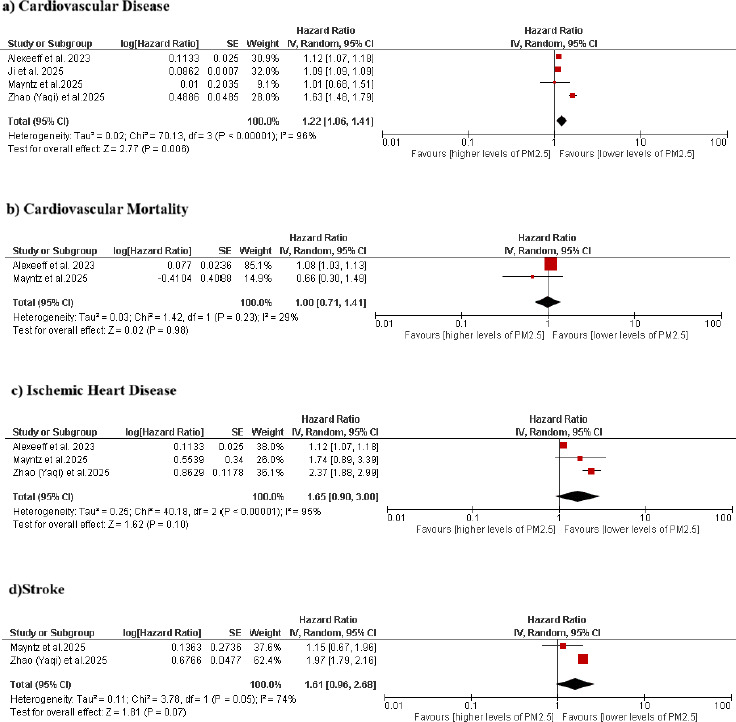
Forest plots showing the associations between long-term PM2.5 exposure and (a) cardiovascular disease, (b) cardiovascular mortality, (c) ischemic heart disease, and (d) stroke.

In the included cohort studies, there was statistically significant difference in the risk of CVD (pooled HR = 1.22, 95% CI 1.06–1.41; p = 0.006), but with a high level of heterogeneity (I^2^ = 96%). On the other hand, there was no significant difference in the occurrence of PM2.5-exposure-associated CVD mortality (pooled HR = 1.00, 95% CI 0.711.41; p = 0.98), and the low heterogeneity was also low (I^2^ = 29%). Regarding ischemic heart disease (IHD), there was a proposed increase in risk, although it was not statistically significant (HR = 1.65, 95% CI = 0.903.00; p = 0.10), and it had high heterogeneity (I^2^ = 95%). In the same way, the risk of stroke had a borderline and non-significant association with increased PM2.5 exposure (HR = 1.61, 95% CI 0.96 2.68; p = 0.07), and with significant heterogeneity (I^2^ = 74%). These results are shown in Figure 2.

### Sensitivity Analysis

The sensitivity analyses were performed to determine the strength of the pooled estimates and the presence of heterogeneity. The leave-one-out analyses showed that in the risk of CVD, the heterogeneity was largely due to the rural Chinese cohort (Zhao et al., 2025) whose removal brought the I^2^ to 0%. Likewise, in case of IHD, by excluding the study by Alexeeff et al., heterogeneity was dropped to 12%. However, sensitivity analysis of CVD mortality and stroke risk showed that the removal of Ji et al., 2025 dropped heterogeneity to 14%.

## Discussion

Four high-quality cohort studies (approximately 9.6 million participants in total) were included in this meta-analysis, which demonstrated that long-term exposure to PM 2.5 positively affected the cardiovascular outcomes and the extent of association varied. The heterogeneity was resolved by sensitivity analysis. The mentioned relationships suggest that PM2.5 is a risk contributor of cardiovascular risks via inflammation, oxidative stress and endothelial dysfunction and linear dose-responses have been identified in the majority of the studies [14-16]. Differences were caused by regional factors: because the sources of particulate matter, including biomass burning, and predispositions, including poor access to healthcare, are likely to be higher in rural regions. The air pollution was also noted higher in Asian cohorts [17, 18]. The European cohorts have also shown the greater association between PM 2.5 levels exposure and CVD [19]. The selection of the multi country analysis proves that the cardiovascular effects, including IHD, have a greater likelihood of being realized in the rural and places with higher pollution. This highlights differences in how vulnerable certain groups are [20]. PM 2.5 exposure has been associated with higher risk of cerebrovascular outcomes including stroke but the heterogeneity was high in our analysis that may be due to small sample size [21].

Our results have been congruent with earlier syntheses that pointed to PM 2.5 being more risky with respect to CVD but disparate compared to the global means. The IHD/stroke associations in Asian studies are better as compared to Western reviews, possibly because of the increased exposures [22]. A Korean cohort study has also shown the association between PM 2.5 and CVD. It has emphasized the role of organic matter in causing endothelial damage; thus leading to cardiovascular mortality [22]. On the other hand, a study from Denmark has shown that long term outcomes are not affected significantly by the exposure of PM 2.5. It has shown that the major adverse cardiovascular events were not affected by chronic exposure of PM 2.5 [11]. But our comprehensive synthesis is against it as the risk of CVD is associated significantly with PM 2.5 exposure.

This has far-reaching consequences on the health care policy and clinical practice of the people. Implementation of more stringent emissions (i.e., the decrease of PM2.5 to less than the levels detected in the areas with the most risks of CVDs, e.g., to levels lower than 5 μg/m^3^ annual mean) may lead to decreased burden of CVD. It will give benefit to particularly those geographic regions where the ensemble models showed greater exposures (e.g., to northwestern and rural China) [8]. The special measures, including clean cooking energy to reduce biomass emissions and green urban spaces to absorb pollution can be disproportionately helpful to those most at risk. This can be viewed as a reaction to environmental hazards [17]. In terms of clinics, spatiotemporal models can be used to include air quality data in the risk assessment of the CVD to enhance the individualized prevention, especially among the elderly or those without any pre-existing CVD [6, 11]. Further, the findings also advance the interdisciplinary solutions, associating epidemiology with the climate policy to remedy the co-benefits of PM2.5 reduction other than the greenhouse gases, which could prevent millions of premature cases of CVD globally [18].

### Limitations

There are several limitations in this study as well. There were only few studies available on this topic and the subgroup analysis was not possible because of the limited data. There was significant heterogeneity in most of the outcomes that undermines the stance of this study. The heterogeneity was due to geographic or methodological differences. Included studies were observational in nature that normally suffer from residual confounding (e.g. diet, genetics).

### Future Directions

The findings of this study has put the emphasis on strict PM2.5 standards and rural programs such as switching to clean energy. Air quality index should be checked regularly as it is a major risk factor of heart diseases. Future studies should track people over a period of time and explore the biological pathway of air pollution. Lifestyle modification will play a key role in reducing this air pollution. Data from Low/middle-income areas will make global policies more reliable.

## Conclusion

This meta-analysis supports that long-term exposure of PM2.5 is related to the higher risks of the occurrence of CVD and the results with ischemic heart disease, cardiovascular mortality and stroke are not statistically significant. The findings support the point that stronger air quality laws, pollution control actions, and enhanced exposure measurements should be used in further large-scale studies. Policy makers should pay attention to this matter and make proper adjustments in management of exposure of PM2.5.

## Figures and Tables

**Table 1:** Baseline characteristics of cohort studies included in the meta-analysis

Study (Year)	Country / Region	Study Design	Study Population	Sample Size	Age Range / Mean Age	Follow-up Duration	PM2.5 Exposure Assessment	Cardiovascular Outcomes
**Alexeeff et al. (2023) [3]**	United States (California)	Retrospective cohort	General adult population (Kaiser Permanente members)	~3.7 million	≥18 years (mean ~41 years)	Up to 10 years	Time-updated residential PM2.5 using ensemble spatiotemporal model	Incident AMI, IHD mortality, CVD mortality
**Ji et al. (2025) [13]**	China (Northwestern regions)	Prospective cohort	Community-based adults without baseline CVD	~5.8 million	≥18 years	~3 years	Spatiotemporal ensemble model (1-km resolution)	Incident CVD
**Zhao et al. (2025) [12]**	China (Rural regions)	Prospective cohort	Rural residents from multicenter cohort	15,502	≥15 years	10 years	High-resolution satellite-based PM2.5 model	Incident CVD, IHD, stroke subtypes
**Mayntz et al. (2025) [11]**	Denmark	Prospective cohort	Older adults from national screening trials	26,723	65–74 years	Up to 10 years (exposure history up to 29 years)	National DEHM/UBM air pollution modeling system	Major adverse cardiovascular events, CVD mortality

**Table 2.** Quality assessment of included cohort studies using the Newcastle–Ottawa Scale

Study (Year)	Selection (max 4)	Comparability (max 2)	Outcome (max 3)	Total NOS Score (max 9)	Risk of Bias
**Alexeeff et al. (2023) [3]**	4	2	3	9	Low
**Ji et al. (2025) [13]**	3	2	2	7	Low
**Zhao et al. (2025) [12]**	4	2	3	9	Low
**Mayntz et al. (2025) [11]**	4	2	2	8	Low
